# Imprinted Genes … and the Number Is?

**DOI:** 10.1371/journal.pgen.1002601

**Published:** 2012-03-29

**Authors:** Gavin Kelsey, Marisa S. Bartolomei

**Affiliations:** 1 Epigenetics Programme, The Babraham Institute, Cambridge, United Kingdom; 2 Centre for Trophoblast Research, University of Cambridge, Cambridge, United Kingdom; 3 Department of Cell and Developmental Biology, University of Pennsylvania Perelman School of Medicine, Philadelphia, Pennsylvania, United States of America; University of Cambridge, United Kingdom

Genomic imprinting in mammals results in the expression of the alleles of a given gene being dependent on their parental origin. Although the existence of imprinted genes was postulated to explain aberrant development of uniparental embryos [1], it wasn't until 1991 that the first imprinted genes were identified by candidate approaches or fortuitously [2]. Given the serious developmental consequences of uniparental embryos, as well as some human syndromes associated with parental-specific deletion of particular chromosome regions, there has been great interest in discovering imprinted genes. As such, several unbiased approaches have been developed in the last 20 years with the goal of obtaining a complete list of imprinted genes. These approaches typically involved identifying genes that were present/absent in complete or partially uniparental embryos, although regions harbouring allele-specific DNA or chromatin modifications have also been used as an indicator of imprinted genes [3]. Earlier studies suggested that imprinted genes likely numbered in the low hundreds. Thus, it was startling to the imprinting community in 2010 when Gregg and colleagues reported >1,000 potential tissue-specific imprinted genes [4]. How could so many have been missed? In fact, others had previously used similar methodology but reported far fewer new imprinted genes [5], [6]. The answer, as discussed in a report from DeVeale and colleagues in this issue of *PLoS Genetics* [7], may not be that so many imprinted genes were missed, but that the limitations of the novel technology may not have been fully appreciated.

**Figure 1:**
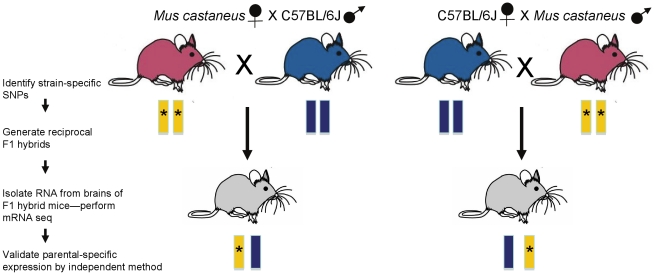
Strategy for the generation and analysis of RNA from F1 hybrid mice. F1 hybrid progeny were generated from reciprocal matings between C57BL/6J and *Mus musculus castaneus* mice. Tissues were isolated from fetal and adult brain of the F1 hybrid mice and mRNA-seq was performed. Using SNPs (asterisks) that were identified in the parental DNA, biases in transcription of parental alleles can be assessed.

The experimental strategy that Gregg et al. and Babak and colleagues [4], [5] used to discover imprinted genes was to perform quantitative, whole-transcriptome sequencing (mRNA-seq) of samples from reciprocal hybrids (fetal or adult brain tissue from F1 hybrid mice, Figure 1) and to identify single nucleotide polymorphisms (SNPs) at which one parental allele is preferentially expressed. Comparison of reciprocal cross samples should rule out genetic effects and mitigate against some experimental noise. The approach is conceptually simple, but it requires robust statistical methods to account for false positives and it is probably fair to say that this remains an area of methodological development.

By reanalysing mRNA-seq datasets from embryonic day 15 (e15) brain published by Gregg et al. [4] and e17.5 brain (their own, [5]), and using the same statistical approach, DeVeale et al. detect similar numbers of known imprinted genes. However, there was far less overlap in the new imprinted genes predicted from the two experiments: each predicted 400–500 candidates, but only about 50 were in common. Although these studies assayed fetal brain from different times, DeVeale and colleagues suspected that the discrepancy was more likely caused by technical issues in generation, mapping, or analysis of the mRNA-seq data.

A prerequisite in analysing large sequence datasets is to know how many candidates could appear “by chance” and to set thresholds to account for this. Although a false discovery rate (FDR) for a dataset can be predicted, there may be sources of experimental noise in the data that are not fully taken into account. Alternatively, it may be possible to determine an FDR empirically. DeVeale and colleagues did so by assuming that SNPs in the same coding exon of an imprinted transcript, but sufficiently distant to be sampled independently, should show the same parental allele expression bias; SNPs discordant in their direction of bias are more likely the consequence of sampling effects at the two positions. Of 1,388 SNP pairs, ∼20% disagreed on direction of bias, suggesting that as many as 40% of the predicted imprinted genes could be false positives. In a second approach, the authors analysed the number of candidates predicted in a “mock reciprocal” cross. This involves taking one F1 mRNA-seq dataset and comparing it with a second F1 dataset as if they were from reciprocal crosses. Worryingly, nearly as many candidate genes emerged from the mock reciprocal as a true reciprocal cross once known imprinted genes had been taken into account.

Using the FDRs determined from mock reciprocal crosses to set a threshold of significance, the authors then reanalysed reciprocal cross mRNA-seq datasets from four tissues: e15 and e17.5 whole brain, adult prefrontal cortex, and preoptic area [4], [5]. They detected 53 putative novel imprinted genes, including three that had already been validated by Gregg et al. Discounting 11 that were associated with known imprinted clusters, 42 candidates remained. They went on to verify a number of transcripts using an independent RT-PCR-based assay, including 17 candidates predicted by Gregg et al. (albeit of the “complex category”, in which there was discordance between parental allele ratios at different SNPs in the same transcript). Six of their 11 candidates validated with parental origin-specific allelic expression bias, but none of the “Gregg candidates” did. Not surprisingly, validation was best in genes with the highest “imprinting score” (a combination of allelic bias and read depth), including genes with biased parental allele expression in multiple samples and concordant at multiple SNPs. These criteria make sense, but such reasoning does not exclude the possibility that there may be additional imprinted genes among the longer candidate lists that exhibit spatiotemporally restricted imprinted expression.

To account for these discrepant findings, DeVeale and colleagues [7] argue that there are potentially multiple sources of systematic error in quantifying allele-specific expression by mRNA-seq, but whether these in aggregate could explain the substantially greater number of candidate imprinted transcripts reported by Gregg et al. is unclear. Nevertheless, the current study demonstrates the importance of appropriate empirically determined FDRs and extensive validation of new candidates by an independent method. Convergent evidence from other datasets, for example, parental-allele-specific DNA methylation or histone modifications, as they become available, will also be useful [8].

Transcriptome sequencing has also been applied to imprinted gene identification in the mouse placenta. The placenta is particularly significant in the physiology of imprinting, owing to its role in regulating fetal growth by controlling the supply of nutrients. In this case, an additional confounding factor is expression of genes in maternally derived cells within the placenta that can remain even after careful dissection [9]. Recently, Okae et al. elegantly demonstrated by embryo transfer that genes highly expressed in contaminating maternal decidual tissue, or other maternal cells infiltrating the placenta, can appear imprinted with maternal-allele-specific expression [10]. Using mRNA-seq of reciprocal hybrid placenta, they identified ∼1,000 genes expressed predominantly from the maternal allele. However, imprinted maternal allele expression was unequivocally demonstrated for only one of 269 genes they sought to verify (in some additional cases, genuine imprinted expression could have been obscured by extremely high expression in contaminating maternal tissue). The success rate for genes with paternal-allele-specific expression was much higher (1/6). This important study casts doubt on a number of apparent imprinted genes previously reported to exhibit maternal-allele-specific expression restricted to the placenta.

Where does this leave us? First, there are probably not more than the few hundred imprinted genes that were predicted many years ago. Moreover, with the rapid development of high-throughput transcriptome sequencing, we have an unprecedented opportunity to identify imprinted genes in any species with a sequenced genome. Thus, understanding the roles of imprinted genes in disease, development, and evolution will be within reach. Nevertheless, the studies by DeVeale and Okae suggest that results from high-throughput screens must be carefully interrogated, including substantial validation by alternative methods.
